# Rational Design of ZnO/Sc_2_CF_2_ Heterostructure with Tunable Electronic Structure for Water Splitting: A First-Principles Study

**DOI:** 10.3390/molecules29194638

**Published:** 2024-09-29

**Authors:** Yong Tang, Yidan Lu, Benyuan Ma, Jun Song, Liuyang Bai, Yinling Wang, Yuanyuan Chen, Meiping Liu

**Affiliations:** 1 School of Energy Engineering, Huanghuai University, Zhumadian 463000, China; 2 Polymer, Recycling, Industrial, Sustainability and Manufacturing (PRISM), Technological University of the Shannon: Midlands Midwest, Athlone, Westmeath N37HD68, Ireland; 3 School of Intelligent Manufacturing, Huanghuai University, Zhumadian 463000, China

**Keywords:** ZnO/Sc_2_CF_2_ heterostructure, electronic structure, band alignment, strain, photocatalytic water splitting

## Abstract

Heterostructures are highly promising photocatalyst candidates for water splitting due to their advanced properties than those of pristine components. The ZnO/Sc_2_CF_2_ heterostructure was designed in this work, and its electronic structure was investigated to explore its potential for water splitting. The assessments of binding energy, phonon spectrum, ab initio molecular dynamics, and elastic constants provide strong evidence for its stability. The ZnO/Sc_2_CF_2_ heterostructure has an indirect band gap of 1.93 eV with a type-Ⅰ band alignment. The electronic structure can be modified with strain, leading to a transition in band alignment from type-Ⅰ to type-Ⅱ. The heterostructure is suitable for water splitting since its VBM and CBM stride over the redox potential. The energy barrier and built-in electric field, resulting from the charge transfer, facilitate the spatial separation of photogenerated carriers, enhancing their utilization efficiency for redox processes. The photogenerated carriers in the heterostructures with lattice compression greater than 6% follow the direct-Z transfer mechanism. The ZnO/Sc_2_CF_2_ heterostructure is confirmed with high photocatalytic activity by a Gibbs free energy change of HER, which is 0.89 eV and decreases to −0.52 eV under an 8% compressive strain. The heterostructure exhibits a remarkable enhancement in both absorption range and intensity, which can be further improved with strains. All these findings suggest that the ZnO/Sc_2_CF_2_ heterostructure is an appreciated catalyst for efficient photocatalytic water splitting.

## 1. Introduction

The pioneering work of Fujishima and Honda on photoelectrochemical water splitting using TiO_2_ electrodes opened a new chapter in solar energy utilization [1]. Producing green hydrogen (H_2_) through photocatalytic water splitting has emerged as a promising way of harnessing solar energy [2]. Photocatalysts, which generate carriers for the redox reactions, have to fulfill some strict requirements. Specifically, the conduction band minimum (CBM) of a photocatalyst must be above the reduction potential (*E*_H+/H2_) of water, while the valence band maximum (VBM) needs to be more negative than the oxidation potential (*E*_O2/H2O_). The high carrier mobility and low carrier recombination rate are also essential to enhance the availability of carriers for redox reactions. Some semiconductors, including BiVO_4_ [3], CdS [4], Ta_3_N_5_ [5], and Cu_2_O [6], have been developed experimentally for photocatalytic water splitting. However, these photocatalysts often suffer from the disadvantages of large band gaps and high carrier recombination rates, which lead to suboptimal photocatalytic performance and limit their practical applicability.

Two-dimensional (2D) materials, such as phosphorenes [7], transition metal dichalcogenides [8], and group-IV monochalcogenides [9], have been reported as photocatalyst candidates due to their tunable electronic structure, large specific surface area, and compositional diversity. Recently, the ZnO monolayer, initially synthesized via vapor deposition [10], has drawn notable attention with its high stability and superior carrier mobility. While the ZnO monolayer has been proposed for photocatalytic water splitting [11], its performance is well below the requirement for practical application due to its wide band gap (~3.4–4.0 eV). In addition, transition metal carbides (MXenes) are another important group of 2D materials with attractive properties [12]. The Sc₂CF₂ monolayer is one of the few MXenes that are semiconducting, making it valuable in energy storage, catalysis, and optoelectronics [13]. Theoretical studies have indicated its exceptional properties in electronic, chemical, and mechanical domains [14,15]. The Sc₂CF₂ monolayer has an appropriate bandgap for photocatalytic water splitting, but its VBM exceeding the *E*_O2/H2O_ hinders its feasibility as a standalone photocatalyst [16]. Thus, suffice it to say that it is essential to implement strategies to tackle the issues of ZnO and Sc_2_CF_2_ monolayers in photocatalytic water splitting.

The emerging 2D heterostructures, formed by stacking different 2D monolayers via van der Waals forces, have enabled the creation of novel and advanced properties [17,18]. Furthermore, the interlayer coupling effect between monolayers allows for tuning the electronic structure [19], improving the interlayer excitonic behavior and boosting the performance. Heterostructures based on 2D materials have shown promising application prospects in fields such as sensors, electronic devices, and energy harvesting and conversion. The bipolar junction transistor device based on an MoTe_2_/GeSe/MoTe_2_ heterostructure has excellent output features with a prompt response against the selective protein, which may be a potential biosensor for detecting target DNA and proteins [20]. Dastgeer reported a photoresponsivity of 3.9 × 10^3^ A*W^−1^ and an external quantum efficiency of 87% for p-GeSe/n-ReS_2_ with a potential for ultra-high-frequency switching applications [21]. Shen prepared the covalent organic frameworks/O-vacancy WO_3_ Z-Scheme Heterostructure with an impressive photocatalytic hydrogen evolution half-rection rate of 593 mmol h^−1^*g^−1^, and this improved performance arises from the intimate electronic coupling at the 2D/2D interface [22]. Previous studies also revealed the significant impact of enhanced interlayer exciton in the interface on improving the solar-to-electricity conversion efficiency of heterostructures [23]. As to water splitting, the heterostructures with a type-II band alignment are highly desirable photocatalysts because the type-II band alignment, with VBM and CBM being located in different 2D materials, spatially reduces the recombination rate of photogenerated carriers. Some ZnO- and Sc_2_CF_2_-based heterostructures have been experimentally and theoretically designed with improved photocatalytic performance. Hezam fabricated the Cs_2_O/Bi_2_O_3_/ZnO heterostructure, which was found to have a type-II band alignment and follow the direct Z-scheme carrier migration pathway [24]. Riffat reported the ZnO/CdTe heterostructure using chemical vapor deposition, finding it with a lower charge transfer resistance and enhanced photocurrent response [25]. Moreover, the MoS_2_/ZnO/WS_2_ [26] and ZnO/Ga_2_SSe [27] heterostructures possess the maximum solar-to-hydrogen (STH) efficiencies of 16.83% and 25.05%, respectively. As to the Sc_2_CF_2_-based heterostructures, the corresponding STH efficiencies of Sc_2_CF_2_/Janus MoSSe [28] and Sc_2_CF_2_/Ti_2_CO_2_ [29] heterostructures are found to be as high as 36.1% and 41.7%, respectively. Furthermore, the electronic structures, band alignments, exciton binding energies, and absorption properties of ZnO- and Sc_2_CF_2_-based heterostructures can also be tuned by the interlayer coupling between the monolayer components [30,31,32,33]. At present, there is no existing report on the ZnO/Sc_2_CF_2_ heterostructure and its potential as a photocatalyst remains unexplored, which is highly expected and of significant interest in developing advanced photocatalysts.

In this work, the ZnO/Sc_2_CF_2_ heterostructure was designed, inspired by the potential of stacking pristine monolayers to enhance photocatalytic performance. A first-principles method was employed to explore its novel properties of structure, stability, electronic structure, band alignment, Gibbs free energy, and absorption, to better understand its photocatalytic performance. The ZnO/Sc₂CF₂ heterostructure with a certain stability owns a type-I band alignment, which is quite sensitive to strain and can be tuned to type-II. The enhanced absorption and excellent photocatalytic water-splitting performance of the ZnO/Sc₂CF₂ heterostructure establish the way for its future application.

## 2. Results and Discussion

The structures of optimized ZnO and Sc_2_CF_2_ monolayers are shown in Figure S1a,c. The results of lattice parameters, obtained using the GGA-PBE functional with DFT-D3, are listed in Table 1. The ZnO monolayer has a graphene-like structure with a lattice constant of 3.29 Å, and the bond length of Zn-O is 2.0 Å. Sc_2_CF_2_ monolayer features a five-layer hexagonal honeycomb structure, with a lattice constant given to be 3.26 Å. The optimized bond lengths of Sc-C and Sc-F are 2.27 Å and 2.21 Å, respectively. The band structures of two monolayers were calculated with HSE06 functional and shown in Figure S1b,d. The ZnO monolayer is a direct semiconductor with a band gap of 3.28 eV while the Sc_2_CF_2_ monolayer has an indirect band gap of 2.09 eV. The VBM and CBM of the ZnO monolayer are −5.81 eV and −2.53 eV, respectively, whereas those of the Sc_2_CF_2_ monolayer are estimated to be −5.56 eV and −3.47eV. These results agree well with previous reports [28,31,34] and validate the theoretical approach used in this work.

**Figure 1:**
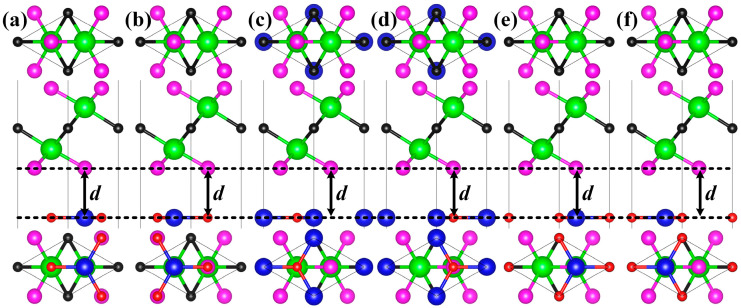
The bottom and side views of ZnO/Sc_2_CF_2_ heterostructures. The red, blue, green, magenta, and black spheres represent the O, Zn, Sc, F, and C atoms, respectively.

The ZnO/Sc_2_CF_2_ heterostructure was created by stacking the Sc_2_CF_2_ monolayer on the ZnO monolayer. There were six possible stacking configurations (SCs) of the ZnO/Sc₂CF₂ heterostructure considered according to the rotation and translation operations on the ZnO monolayer, as illustrated in Figure 1a–f. The results of lattice constant *a*, interlayer distance *d*, binding energy *E*_b_, and band gap *E*_g_ of all six heterostructures are listed in Table 2. The lattice constants of six entirely relaxed ZnO/Sc_2_CF_2_ heterostructures are very close, ranging from 3.26 Å to 3.37 Å. The SC-Ⅰ ZnO/Sc_2_CF_2_ heterostructure owns the shortest interlayer distance of 2.93 Å, while the largest distance of 3.42 Å is observed in the SC-Ⅱ heterostructure. To determine the most stable SC, the values of *E*_b_ for six heterostructures were calculated with the following expression:

(1) $$E_{b}=\frac{E_{\mathrm{het}}\;-{\;E}_{\mathrm{ZnO}}\;-E_{\mathrm{Sc}2\mathrm{CF}2}}{S}$$

where *E*_het_, *E*_ZnO_, and *E*_Sc2CF2_ indicate the energies of heterostructures, ZnO monolayer, and Sc_2_CF_2_ monolayer, respectively, while *S* means the interface area. All the values of *E*_b_ for ZnO/Sc_2_CF_2_ heterostructures are negative, promising the feasibility of their experimental creation. The six values are comparable to those of vdW heterostructures, indicating that ZnO and Sc_2_CF_2_ are bonded together through the vdW forces [35]. The SC-Ⅰ heterostructure exhibits the most negative *E*_b_ of −34.59 meV*Å^−2^, confirming its optimal energetic stability. Therefore, subsequent research primarily focuses on this structure.

**Figure 2:**
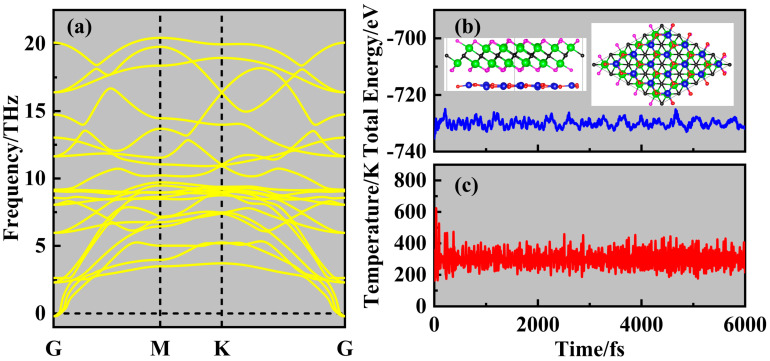
(**a**) The phonon spectrum of the ZnO/Sc_2_CF_2_ heterostructure. The variations in (**b**) energy and (**c**) temperature of the ZnO/Sc_2_CF_2_ heterostructure during the AIMD simulation at 300 K; the insert shows its final snapshot.

The phonon spectrum of the ZnO/Sc_2_CF_2_ heterostructure was then calculated using the 3 × 3 × 1 supercell to assess its dynamic stability. From Figure 2a, there are only a few negligible imaginary frequencies seen near the G-point, similar to those found in the phonon spectra of some experimentally prepared 2D materials [36,37,38]. The observation of negligible imaginary frequencies is attributed to limited computational accuracy and can be eliminated with higher accuracy parameters or larger supercells. Therefore, the ZnO/Sc_2_CF_2_ heterostructure is dynamically stable. The NVT-ensembled and NPT-ensembled ab initio molecular dynamics (AIMD) simulations were carried out on a 4 × 4 × 1 supercell to validate the thermodynamic stability of the ZnO/Sc_2_CF_2_ heterostructure. As shown in Figure 2b, the total energy of the ZnO/Sc_2_CF_2_ heterostructure undulates slightly, and no significant structural distortion is noticed in the final snapshot after the heating process, confirming its thermodynamic stability at the temperature of 300 K. In the NPT-AIMD simulation, the ambient pressure and room temperature were considered, and its results in Figure S2 indicate that the lattice constant obtained from the NPT-AIMD simulation is close to four times that of the ZnO/Sc_2_CF_2_ heterostructure. There are only minor changes in the lattice constant throughout the simulation process and the final snapshot of the ZnO/Sc_2_CF_2_ heterostructure maintains good structural integrity. Comparing the results of NVT-AIMD and NPT-AIMD, there are no significant changes observed in the two final snapshots after pressure is considered. These findings of NPT-AIMD simulation further confirm the thermodynamic stability of ZnO/Sc_2_CF_2_ under real-world conditions. Furthermore, the elastic constants of the ZnO/Sc_2_CF_2_ heterostructure were evaluated with the energy–strain method. The independent elastic constants *C*_11_ and *C*_12_ for the ZnO/Sc_2_CF_2_ heterostructure are 274.4 N/m and 90.7 N/m, respectively. The results of *C*_11_ and *C*_12_ satisfy the elastic stability criteria [39], further confirming the mechanical stability of the ZnO/Sc_2_CF_2_ heterostructure. To sum up, these findings have comprehensively authorized the stability of the SC-Ⅰ ZnO/Sc_2_CF_2_ heterostructure, indicating its potential for experimental realization in the future.

**Figure 3:**
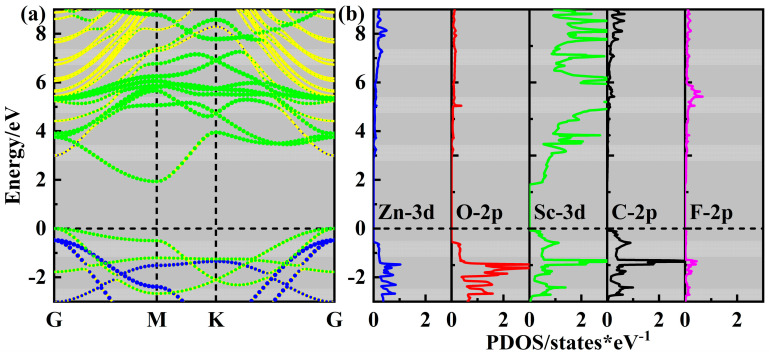
(**a**) The projected band structure and (**b**) PDOS of the SC-Ⅰ ZnO/Sc_2_CF_2_ heterostructure.

The projected band structures and density of states (PDOS) of all six ZnO/Sc_2_CF_2_ heterostructures were calculated using HSE06 functional to explore their electronic properties. The band structures and PDOS are shown in Figure 3 and Figure S3, and the values of *E*_g_ are given in Table 2. The band structures reveal that all ZnO/Sc_2_CF_2_ heterostructures are indirect semiconductors, with the VBM and CBM exits in the G and M points, respectively. Both the VBM and CBM are located in the Sc_2_CF_2_ layer, which means these heterostructures possess the type-Ⅰ band alignment, which is primarily due to the results in Table 1 that the band edges of the Sc_2_CF_2_ monolayer are enveloped by those of the ZnO monolayer. According to the analysis of PDOS, the VBM is predominantly derived from the Sc-3d and C-2p orbitals, while the CBM almost entirely originates from the Sc-3d orbital. As to the results of *E*_g_, the SC-Ⅰ ZnO/Sc_2_CF_2_ heterostructure owns the largest value of 1.93 eV, and those of the other five range from 1.90 eV to 1.92 eV. The values of *E*_g_ for ZnO/Sc_2_CF_2_ heterostructures are lower than those of the pristine ZnO and Sc_2_CF_2_ monolayers, and this reduction may be attributed to the interlayer coupling effect between the ZnO and Sc_2_CF_2_ layers in the heterostructures. The above results indicate that the SC is insignificant in determining the band structure and *E*_g_ for ZnO/Sc_2_CF_2_ heterostructures, which improves the feasibility of desired results in electronic properties experimentally.

Strain is a common interlayer effect in 2D heterostructures, with their electronic properties being highly sensitive to it. Hence, the biaxial strain *ε* has been adopted to tune the electronic properties of the ZnO/Sc_2_CF_2_ heterostructure, with the *ε* defined as:

(2) $$\varepsilon \;₌\;\frac{a{\;-\;a}_{0}}{a_{0}}$$

**Figure 4:**
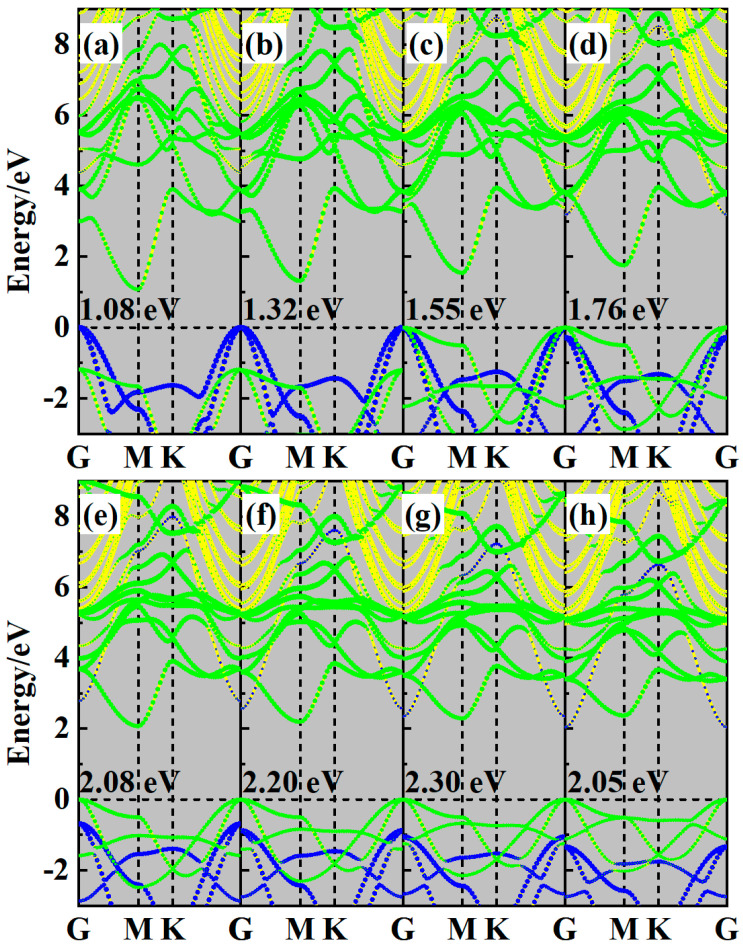
The projected band structures of (**a**) −8%, (**b**) −6%, (**c**) −4%, (**d**) −2%, (e) 2%, (**f**) 4%, (**g**) 6%, and (**h**) 8% strained ZnO/Sc_2_CF_2_ heterostructures. The insert values represent the corresponding *E*_g_ for strained heterostructures.

In this expression, *a* and *a*_0_ present the lattice constants of strained and freestanding heterostructures, respectively. A total of eight strain values were considered, with the value of *ε* ranging from the smallest compressive strain of −8% to the largest tensile strain of +8%. The band structures of strained ZnO/Sc_2_CF_2_ heterostructures are shown in Figure 4. It is evident from the values in the band structures that lattice compression generally reduces the *E*_g_ of the ZnO/Sc_2_CF_2_ heterostructure, except for the 8% tensile strain. When its lattice is compressed by 8%, the *E*_g_ of the ZnO/Sc_2_CF_2_ heterostructure significantly decreases to 1.08 eV. The value of *E*_g_ for the ZnO/Sc_2_CF_2_ heterostructure increases as the lattice compression decreases, reaching 2.3 eV at a 6% tensile strain. Within the strain range of −8% to +6%, the ZnO/Sc_2_CF_2_ heterostructure remains an indirect bandgap semiconductor, with the VBM and CBM located at the Γ and M points, respectively. The effect of strain on the VBM is more significant. Specifically, with lattice compression, the VBM of the ZnO layer increasingly surpasses that of the Sc_2_CF_2_ layer. When compressed by 4%, the VBM is primarily contributed by the ZnO layer, as the CBM is derived from the Sc_2_CF_2_ layer. The change in the VBM results in a transition of band alignment type for the ZnO/Sc_2_CF_2_ heterostructure from type-Ⅰ to type-Ⅱ. When the lattice of the ZnO/Sc_2_CF_2_ heterostructure transitions from compression to tension, the effect of strain on the CBM is that the CBM of the ZnO layer gradually approaches that of the Sc_2_CF_2_ layer. When the lattice is stretched by 8%, the CBM of the ZnO layer falls below that of the Sc_2_CF_2_ layer, which forms a type-II band alignment. For the ZnO/Sc_2_CF_2_ heterostructure with 8% tensile strain, its CBM shifts from the Sc_2_CF_2_-occupied M-point to the G-point, which is contributed by the ZnO layer. The shifting in CBM tunes the ZnO/Sc_2_CF_2_ heterostructure to be a direct semiconductor, with the *E*_g_ dropping to 2.05 eV. Although both compressive and tensile strains can tune the band alignments of the ZnO/Sc_2_CF_2_ heterostructures to type-Ⅱ, the orbitals occupying the VBM and CBM shift. Due to the charge transfer induced by interlayer coupling, a built-in electric field (*E*_in_) will be generated in the interface. The *E*_in_ will result in different reaction mechanisms in the ZnO/Sc_2_CF_2_ heterostructures, which have the strain-induced type-Ⅱ band alignment. All these above-mentioned outcomes indicate that the varied effects of strain enable the experimental tuning of the electronic structure for the ZnO/Sc_2_CF_2_ heterostructure, which is beneficial and crucial for optimizing and boosting its photocatalytic performance.

The charge transfer induced by interlayer coupling in the heterostructures plays a considerable role in their photocatalytic performance. Once the two monolayers come into contact, the difference in the work function *W*_f_ will excite the electron transfer in the ZnO/Sc_2_CF_2_ heterostructure. The *W*_f_ is the difference between the vacuum level (*E*_vac_) and Fermi level (*E*_F_) and is given as below:

(3) $$W\;₌\;E_{\mathrm{vac}}\;-E_{F}$$

**Figure 5:**
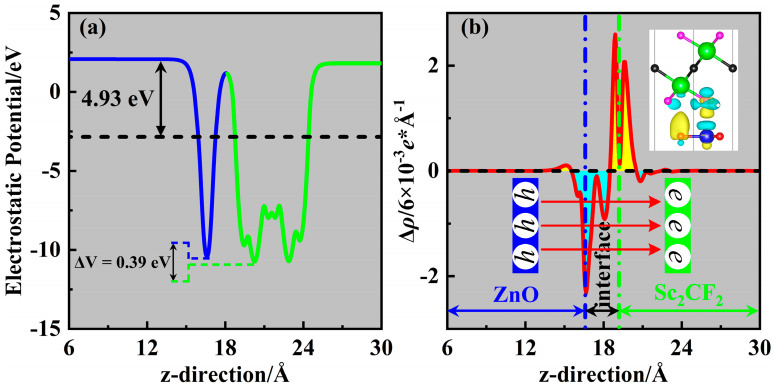
(**a**) The electrostatic potential of the ZnO/Sc_2_CF_2_ heterostructure. (**b**) The planar-averaged charge density difference Δρ for the ZnO/Sc_2_CF_2_ heterostructure; the insert is the side view of the charge density difference with its isosurface value of 3 × 10^−4^ e*Å^−3^.

From the potential energies shown in Figure S4, the corresponding values of *W*_f_ are 4.79 eV and 5.02 eV for the ZnO and Sc_2_CF_2_ monolayers, respectively. Thus, electrons will flow from the ZnO layer to the Sc_2_CF_2_ layer when their contact is established. The migration of electrons causes the increase in the Fermi level of ZnO, while that of Sc_2_CF_2_ decreases. From Figure 5a, the *W*_f_ of ZnO/Sc_2_CF_2_ heterostructure is given as 4.93 eV when the two Fermi levels reach equilibrium. Furthermore, the Bader charge analysis [40] indicates that the Sc_2_CF_2_ layer gains 0.011 electrons from the ZnO layer. A potential drop of 0.39 eV is observed across the interface, and this drop forms the *E*_in_ originating from the ZnO layer toward the Sc_2_CF_2_ layer. The resulting *E*_in_ facilitates the separation of photogenerated electrons and holes, enabling them to be effectively utilized in photocatalytic redox processes.

To further explore the charge transfer behavior in the ZnO/Sc_2_CF_2_ heterostructure, the charge density difference Δ*ρ* was obtained using the following Formula (4):

(4) $$∆\rho \;₌\;\rho_{\mathrm{het}}-\;\rho_{\mathrm{ZnO}}\;-\rho_{\mathrm{Sc}2\mathrm{CF}2}$$

Here, *ρ*_het_, *ρ*_ZnO_, and *ρ*_Sc2CF2_ stand for the corresponding charge densities of the ZnO/Sc_2_CF_2_ heterostructure and the two pristine monolayers. Because of the previous difference in *W*_f_ between the ZnO and Sc_2_CF_2_ monolayers, both the 2D and 3D views of Δ*ρ* present in Figure 5b illustrate the occurrence of electron depletion and accumulation in the interface. Yellow and cyan regions in Figure 5b represent the areas of electron accumulation and depletion, respectively. It can be observed that electrons accumulate on the Sc_2_CF_2_ layer while being depleted on the ZnO layer. This result is the formation of hole-rich and electron-rich regions close to the ZnO layer and Sc_2_CF_2_ layer, respectively. Thus, the electron migration is reasonable for the 0.39 eV potential drop exhibited in Figure 5a. Furthermore, the pronounced strain effects on Δ*ρ* for the strained ZnO/Sc_2_CF_2_ heterostructures are noticed in Figures S5 and S6, reproducing the previously discussed tuning effect of strains on the electronic structures.

**Figure 6:**
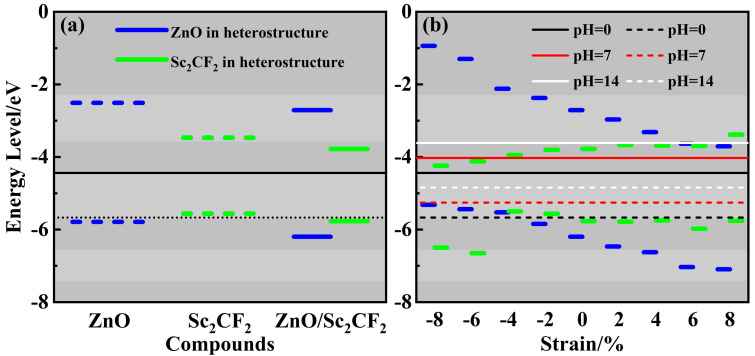
(**a**) The band edge positions of the ZnO/Sc_2_CF_2_ heterostructure, as well as those of pristine monolayers. (**b**) The band alignments of strained ZnO/Sc_2_CF_2_ heterostructures. The solid and dashed lines are the oxidation and reduction potentials, respectively.

A suitable band edge position is one of the prerequisites for photocatalysts used for water splitting. The band edges of the ZnO/Sc_2_CF_2_ heterostructure, as well as those of the ZnO and Sc_2_CF_2_ monolayers, have been calculated to examine their photocatalytic activities. As depicted in Figure 6a, the band edges of the pristine ZnO monolayer straddle the water redox potential at pH = 0. The CBM level of the ZnO monolayer is significantly beyond the *E*_H+/H2_, suggesting its strong reduction ability to obtain a high hydrogen evolution reaction (HER) performance. However, the VBM of ZnO is only marginally lower than the *E*_O2/H2O_, which indicates its poor oxygen evolution reaction (OER) activity. The Sc_2_CF_2_ monolayer is gifted with HER activity due to its CBM being well above the *E*_H+/H2_, but it is unsuitable for the OER process since its VBM exceeds the *E*_O2/H2O_. For the ZnO/Sc_2_CF_2_ heterostructure, the VBM and CBM, which are entirely contributed by the Sc_2_CF_2_ layer, straddle the water redox potential. The interlayer coupling is likely responsible for the downward shift of the VBM in the Sc_2_CF_2_ layer. This suggests that the heterostructure can potentially facilitate both the HER and OER. Overall, the ZnO/Sc_2_CF_2_ heterostructure exhibits a significantly reduced *E*_g_ compared to the ZnO monolayer and a more suitable VBM position than the Sc_2_CF_2_ monolayer. Therefore, the ZnO/Sc_2_CF_2_ heterostructure serves as a more promising photocatalyst candidate for water splitting compared to either of the individual monolayers. The band edge positions and band alignments of strained ZnO/Sc_2_CF_2_ heterostructures are also evaluated and plotted in Figure 6b. All the strained ZnO/Sc_2_CF_2_ heterostructures maintain photocatalytic water-splitting activity all over the wide pH ranges. Overall, the effects of strain on the band edge positions of the ZnO and Sc_2_CF_2_ layers are opposite. As the lattice of the ZnO/Sc_2_CF_2_ heterostructure is compressed, the band edge positions of the ZnO layer gradually shift upward, while the VBM and CBM positions of the Sc_2_CF_2_ layer decrease. This leads to a transition in the band alignment of the ZnO/Sc_2_CF_2_ heterostructure from type-I to type-II when it is compressed by 4%. In the stretched ZnO/Sc_2_CF_2_ heterostructure, the VBM and CBM of the ZnO layer move towards lower energy levels, while those of the Sc_2_CF_2_ layer move upwards. When the lattice is stretched by 8%, the band alignment transitions from type-I to type-II again. The type-II band alignments of compressed and stressed ZnO/Sc_2_CF_2_ heterostructures can spatially separate the photo-generated electrons and holes to improve the efficiency of photocatalytic reactions. The recombination and migration behavior of photogenerated carriers in these two type-II band alignments are different, which leads to different reaction mechanisms.

**Figure 7:**
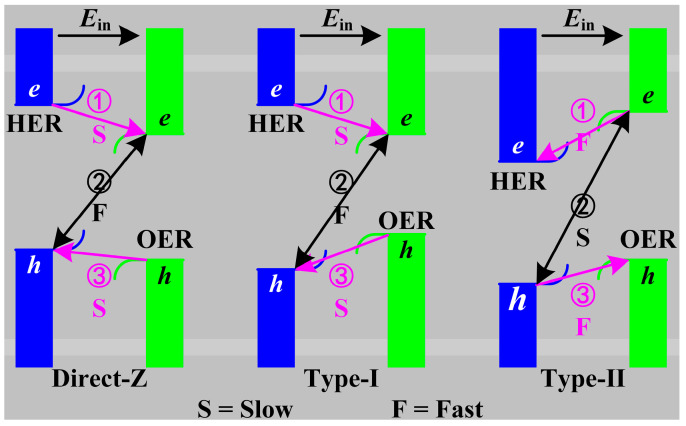
The photocatalytic mechanism in ZnO/Sc_2_CF_2_ heterostructures.

The photocatalytic mechanisms of the ZnO/Sc_2_CF_2_ heterostructures are illustrated in Figure 7. As previously mentioned, when the two monolayers come into contact, charge transfer occurs in the interface, leading to the ZnO layer becoming positively charged and the Sc₂CF₂ layer being negatively charged. The charge transfer results in the energy bands in the ZnO layer bending upward whereas those in the Sc₂CF₂ layer bend downward, as displayed in Figure 7. The band bending of the two layers generates extra potential barriers. When the ZnO/Sc_2_CF_2_ heterostructure is illuminated, electrons are excited to the CBM of ZnO and Sc_2_CF_2_, leaving holes in the VBM. Then, the carrier transfer, occurring in the ZnO/Sc_2_CF_2_ heterostructures between the ZnO and Sc_2_CF_2_ layers, follows three main pathways [28]: ① electrons transfer between the CBM of ZnO and CBM of Sc_2_CF_2_; ② the recombination of the electron-hole between the VBM of ZnO and the CBM of Sc_2_CF_2_ (the CBM of ZnO and the VBM of Sc_2_CF_2_ in a type-Ⅱ alignment); ③ holes flow from the VBM of Sc_2_CF_2_ to the VBM of ZnO (the VBM of ZnO to the VBM of Sc_2_CF_2_ in a type-Ⅱ alignment). The diffusion (① and ③) and the recombination (②) are competing paths for photo-generated carriers.

ZnO/Sc_2_CF_2_ heterostructures with strains ranging -from 4% to 6% have the type-Ⅰ band alignments, and the path ① is impeded because of the potential barrier in the CBM of the ZnO layer, leaving the electrons on the CBM of ZnO. Paths ② and ③ are promoted due to the *E*_in_, leading the holes mainly retained on the VBM of the Sc_2_CF_2_ layer. Thereby, in the ZnO/Sc_2_CF_2_ heterostructures with type-Ⅰ band alignments, the HER and OER are highly likely to proceed in the CBM of the ZnO layer and the VBM of the Sc_2_CF_2_ layer, respectively. When the lattice of the heterostructure lattice is compressed by 6% or even more, the band alignment changes to a type-Ⅱ. The paths ① and ③ are hindered by the potential barriers and *E*_in_, respectively, while the *E*_in_ facilitates the carrier recombination in path ②. The photocatalysis of these compressed ZnO/Sc_2_CF_2_ heterostructures follows the direct-Z mechanism in the all-pH-range, which breaks through the limitation of a photocatalyst with an *E*_g_ not less than 1.23 eV and has strong redox ability. Ultimately, the electrons on the CBM in ZnO and the holes on the VBM in Sc_2_CF_2_ are reserved for the HER and OER, respectively. If the lattice is stretched by 8%, the ZnO/Sc_2_CF_2_ heterostructure exhibits a type-Ⅱ band alignment with ZnO and Sc_2_CF_2_ occupying the CBM and VBM, respectively, which is different from the type-Ⅱ band alignments of compressed heterostructures. In the stretched heterostructure, the *E*_in_ accelerates the carrier diffusion along the paths ① and ③, making these processes more prominent compared to the recombination occurring along path ②. Therefore, electrons and holes finally accumulate in the CBM of ZnO and the VBM of Sc_2_CF_2_, respectively, where they participate in the subsequent redox reactions. Based on the above discussion, although the strained ZnO/Sc_2_CF_2_ heterostructures are different in the band alignment type and carrier transfer mechanism, the comprehensive effects of the energy barrier and the *E*_in_ ensure that photogenerated electrons and holes are significantly separated and retained in the CBM of ZnO and the VBM of Sc_2_CF_2_, respectively. This spatial separation is beneficial to enhancing the utilization of photogenerated electrons and holes, thereby improving the photocatalytic performance. From the perspective of a photocatalytic mechanism, the ZnO/Sc_2_CF_2_ heterostructures may be more advanced than pristine monolayers in water splitting.

Superior absorption behavior is one of the crucial properties of photocatalysts since it rules the upper limitation of photogenerated carriers available for follow-up HER and OER processes. The absorption coefficients of the ZnO/Sc_2_CF_2_ heterostructures and the pristine components have been calculated with the Equation (5) [41]:

(5) $$\alpha \left(\omega \right)=\sqrt{2}\omega \sqrt{\sqrt{\varepsilon_{1}^{2}\left(\omega \right)+\varepsilon_{2}^{2}\left(\omega \right)}\;-\;\varepsilon_{1}(\omega )}$$

**Figure 8:**
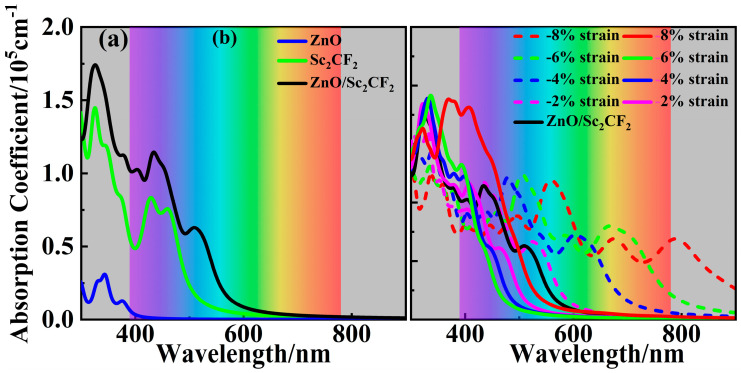
(**a**) The absorption coefficients of the ZnO/Sc_2_CF_2_ heterostructure and two freestranding components. (**b**) The absorption coefficients of strained ZnO/Sc_2_CF_2_ heterostructures.

In this equation, the ω expresses the photon frequency, whereas the *ε*_1_(ω) and *ε*_2_(ω) are the real and imaginary parts of the dielectric function. From the results of the absorption coefficient in Figure 8a, the large *E*_g_ of the ZnO monolayer makes it primarily suitable for absorbing ultraviolet light, and its absorption intensity is relatively weak. The absorption performance of the Sc_2_CF_2_ monolayer is more conspicuous than that of the ZnO monolayer, as its smaller *E*_g_ consents it to absorb in a broader region with a higher intensity. The ZnO/Sc_2_CF_2_ heterostructure is granted a much broader absorption range, extending into the infrared region, for its further smaller *E*_g_ compared to the two pristine monolayers. More importantly, the absorption intensity of the ZnO/Sc_2_CF_2_ heterostructure is significantly enhanced, likely attributed to the interlayer coupling effect boosting the optical excitation [42]. To reveal the strain effect on the absorption property, the absorption coefficients of strained ZnO/Sc_2_CF_2_ heterostructures were evaluated and are presented in Figure 8b. As mentioned earlier, compressive strains reduce the *E*_g_ of ZnO/Sc_2_CF_2_ heterostructure to broaden the absorption range, while the enlargement of *E*_g_ for stretched heterostructures narrows the range. Especially for ZnO/Sc_2_CF_2_ heterostructures with compressive strains of 4%, 6%, and 8%, their absorption performance in the visible region is notably enhanced. The absorption intensities of the strained ZnO/Sc_2_CF_2_ heterostructures have been quite improved for the more remarkable interlayer coupling effects introduced by strains. Notably, the intensity of the ZnO/Sc_2_CF_2_ heterostructure stretched by 8% is appreciable because of its direct bandgap. All the ZnO/Sc_2_CF_2_ heterostructures are superior to the two pristine monolayers in both absorption range and intensity, making them highly advantageous for efficient photocatalytic water splitting.

**Figure 9:**
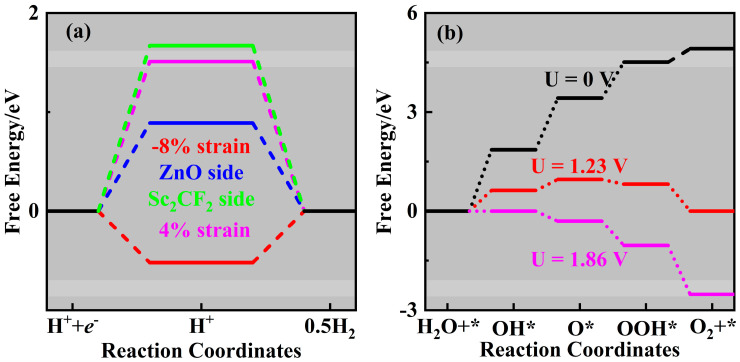
Gibbs free energies for the (**a**) HER and (**b**) OER occurred in ZnO/Sc_2_CF_2_ heterostructures.

The Gibbs free energy changes (Δ*G*) in the HER and OER are estimated from a thermodynamic perspective to explore the feasibility of water splitting on the ZnO/Sc_2_CF_2_ heterostructure [43]; the detailed computational methods have been presented in previous work [44]. The 4 × 4 × 1 supercells of the unstrained ZnO/Sc_2_CF_2_ heterostructure and those with −8% and +4% strains have been employed to absorb the intermediates, The results of Δ*G* for the HER and OER are shown in Figure 9a,b, respectively, while the most energetically stable absorb configurations are displayed in Figures S7 and S8. For the unstrained ZnO/Sc_2_CF_2_, the H-atom was considered to absorb on both the ZnO side and the Sc_2_CF_2_ side. When the H-atom absorbs on the ZnO side, the value of Δ*G* gives 0.89 eV, whereas that of the H-atom on the Sc_2_CF_2_ side is 1.67 eV. This indicates that the HER is favored to occur on the ZnO side, which may be because of the high energy level of its VBM. Our previous work calculated the *p*-band center to confirm that the strong binding between the H-atom and F-atom should be the reason for the higher value on the Sc_2_CF_2_ side [45]. Therefore, the HER is considered to occur on the ZnO side in the strained heterostructures to evaluate Δ*G*. The values of Δ*G* for heterostructures with −8% and +4% strains are determined to be −0.52eV and 1.54 eV, respectively. This variation is attributed to the combined effects of obvious interlayer charge transfer and the regulation of band edge positions, which are caused by strains in ZnO/Sc_2_CF_2_ heterostructures. Considering the values of Δ*G* for the HER proceeding on ZnO/Sc_2_CF_2_ heterostructures are smaller or comparable to those of the experimental and theoretically reported N-Ni_3_S_2_/NF (2.63 eV), g-ZnO/PtSe_2_ (3.719 eV), and g-C_3_N_4_/ZnO (1.09 eV) heterostructure photocatalysts [46,47,48], the O-2p orbital distributions of H-adsorbed ZnO/Sc_2_CF_2_ heterostructures shown in Figure S9 was provided to further comprehend the difference in the HER performance for the 8% compressed, free, and 4% stressed ZnO/Sc_2_CF_2_ heterostructures. The values of the O-2p band center for 8% compressed, free, and 4% stressed ZnO/Sc_2_CF_2_ heterostructures are −0.905 eV, −0.535 eV, and −0.398 eV, respectively. A lower band center means a stronger adsorption strength of H on the 8% compressed ZnO/Sc_2_CF_2_ heterostructure, which is conducive to a higher photocatalytic performance [49]. From the point of low value of Δ*G*, the HER with the ZnO/Sc_2_CF_2_ heterostructures should likewise be experimentally practicable with a favorable performance. As discussed previously, the OER tends to occur on the Sc_2_CF_2_ side, and the values of Δ*G* for four reaction steps are 1.86 eV, 1.56 eV, 1.09 eV, and 0.41 eV, respectively. The four reaction steps involved in the OER are uphill, and the first step reaction to form the OH is the rate-limiting step. The Δ*G* of the rate-limiting step is much lower than those of previously proposed g-ZnO/PtSe_2_ (5.358 eV), MoS_2_/g-C_3_N_4_ (3.75 eV), InSe/*g*-C_3_N_4_ (4.514 eV), CrS_3_/GeSe (2.61 eV) heterostructures [47,50,51,52], indicating its higher activity in catalyzing the OER possesses. When an external potential U of 1.23 eV is applied, the overpotential of the OER reduces to 0.63 eV, which means the overpotential of 0.63 V for the OER. This finding of the ZnO/Sc_2_CF_2_ heterostructure is more positive than those of MoSSe/blue phosphorene and PtS_2_/GaSe heterostructures with high STH efficiencies [53,54]. Once the U reaches 1.86 eV, all reaction steps go downhill, entirely overcoming the overpotential required for the OER. Consequently, the ZnO/Sc_2_CF_2_ heterostructures with appropriate Δ*G* exhibit attractive application potential as photocatalysts for water splitting to produce clean H_2_ energy.

## 3. Computational Method

A Vienna ab initio simulation package (VASP) [55], based on the density functional theory, was employed to perform the theoretical calculations. The generalized gradient approximation within the Perdew–Burke–Ernzerhof (GGA-PBE) scheme was used to define the exchange–correlation functional [56]. Projector-augmented wave pseudopotentials (PAW) were adopted [57,58], as the cutoff energy was set to 500 eV. The structures of the ZnO/Sc_2_CF_2_ heterostructure and the two pristine monolayers were completely optimized using the GGA-PBE functional until the total energy and force converged to less than 10^−5^ eV and 0.01 eV*Å^−1^, respectively. The Heyd–Scueria–Ernzerhof hybrid functional (HSE06) [59] was inserted to conduct the calculations of the band structure and absorption coefficient. The van der Waals interactions in the ZnO/Sc_2_CF_2_ heterostructure were expressed using the DFT-D3 approach [60]. A vacuum space of 30 Å was set in the z-direction for all 2D compounds to prevent interactions between neighboring layers. Gamma-centered k-points [61] with grids of 15 × 15 × 1 and 21 × 21 × 1 were applied to sample the Brillouin zone for structure optimization and property computations. To access the thermal stability of the ZnO/Sc_2_CF_2_ heterostructure, a 4 × 4 × 1 supercell was created for the NVT-ensembled AIMD simulation [62] using the algorithm of the Nosé–Hoover thermostat. The simulation was conducted at 300 K for 6 ps, with a time step of 1 fs. The phonon spectrum of ZnO/Sc_2_CF_2_ was calculated with a PHONOPY code based on density functional perturbation theory (DFPT) [63]. The method proposed by Toroker et al. [64] was employed to determine the band edge position. The pre- and post-visualization were operated with a VASPKIT package [65] and VESTA code [66].

## 4. Conclusions

In this work, the electronic structure and photocatalytic property of the novel proposed ZnO/Sc_2_CF_2_ heterostructure have been investigated with a first-principles method. The heterostructure with sizable stability possesses a type-I band alignment, and its band gap is reduced compared with the components. The electronic structure of the ZnO/Sc_2_CF_2_ heterostructure is sensitive to strain, which not only regulates the band gap but also tunes its band alignment to type-II. The band edge positions of the ZnO/Sc_2_CF_2_ heterostructure are suitable for photocatalytic water splitting, while the capabilities of photocatalytic water splitting for strained heterostructures are kept over a wide strain range. The energy barrier and built-in electric field result in the photogenerated electrons and holes retaining in the CBM of ZnO and the VBM of Sc_2_CF_2_, respectively. In addition, the ZnO/Sc_2_CF_2_ heterostructure exhibits enhanced absorption performance and appropriate Gibbs free energies. All the findings about band gap, band edge position, absorption, and Gibbs free energy suggest that the ZnO/Sc_2_CF_2_ heterostructure is a promising photocatalyst.

## Figures and Tables

**Table 1:** Results of lattice constant *a* (Å), bond length *l* (Å), band gap *E*_g_ (eV), band edge (VBM and CBM) positions (eV), and work function *W*_f_ (eV) for ZnO and Sc_2_CF_2_ monolayers.

Items	*a*	*l*_Zn-O_	*l*_Sc-C_	*l*_Sc-F_	*E*_g_	VBM	CBM	*W*_f_
ZnO	3.29	2.0	-	-	3.28	−5.79	−2.51	4.82
Sc_2_CF_2_	3.22	-	2.27	2.21	2.09	−5.56	−3.47	5.02

**Table 2:** Results of lattice constant *a* (Å), interlayer distance *d* (Å), binding energy *E*_b_ (meV*Å^−2^), band gap *E*_g_ (eV) for ZnO/Sc_2_CF_2_ heterostructures.

SC	SC-I	SC-II	SC-III	SC-IV	SC-V	SC-VI
*a*	3.27	3.26	3.27	3.27	3.27	3.26
*d*	2.93	3.42	3.12	3.32	3.01	3.24
*E*_b_	−34.59	−19.14	−31.19	−19.89	−28.28	−26.22
*E*_g_	1.93	1.90	1.92	1.90	1.92	1.90

## Data Availability

Data will be made available on request.

## Supplementary Materials

The following supporting information can be downloaded at: https://www.mdpi.com/article/10.3390/molecules29194638/s1, Figure S1: The 2 × 2 × 1 supercells of (a) ZnO and (c) Sc2CF2 monolayers. The corresponding band structures are shown in (b,d), respectively; Figure S2: The evolution of lattice constant a for the 4 × 4 × 1 supercell of ZnO/Sc2CF2 heterostructure in the NPT-AIMD simulation at ambient pressure and room temperature, and the inserts are the top, side, and bottom views for its final snapshot at the end of NPT-AIMD simulation; Figure S3: The projected band structures and dos of (a) SC-Ⅱ, (b) SC-Ⅲ, (c) SC-Ⅳ, (d) SC-Ⅴ, and (e) SC-Ⅵ ZnO/Sc2CF2 heterostructures; Figure S4: The potential energies of (a) ZnO and (b) Sc2CF2 monolayers; Figure S5: The planar-averaged charge density difference Δρ for the strained and free-standing ZnO/Sc2CF2 heterostructures; Figure S6: The charge density difference with isosurface value of 3×10-4 e*Å-3 for ZnO/Sc2CF2 heterostructures with (a) −8%, (b) −6%, (c) −4%, (d) −2%, (e) 0%, (f) 2%, (g) 4%, (h) 6%, (i) 8% strain, respectively;Figure S7: The top views of H-adsorption configurations on ZnO/Sc2CF2 heterostructure: (a) H on the ZnO side, (b) H on the Sc2CF2 side; Figure S8: The top views of ZnO/Sc2CF2 heterostructure with absorbed intermediates: (a) OH*, (b) O*, and (c) OOH*; Figure S9: The O-2p orbital distribution of H-adsorbed ZnO/Sc2CF2 heterostructures.
