# Supplementary material for: Rational Design of ZnO/Sc2CF2 Heterostructure with Tunable Electronic Structure for Water Splitting: A First-Principles Study
Source: Molecules. 2024 Sep 29;29(19):4638. doi: 10.3390/molecules29194638 (PMC11477741; doi:10.3390/molecules29194638)
Supplement: Supplementary file 1 [file molecules-29-04638-s001.zip › molecules-3224085-supplementary.pdf]

Rational design of ZnO/Sc_2CF_2 heterostructure with tunable electronic structure for water-splitting: A first-principles study

Yong Tang[1,2], Yidan Lu[1], Benyuan Ma[1], Jun Song[1], Liuyang Bai[1], Yinling Wang[1], Yuanyuan Chen[2*], Meiping Liu[3*].

1. School of Energy Engineering, Huanghuai University, Zhumadian, Henan 463000, China;

2. Polymer, Recycling, Industrial, Sustainability and Manufacturing (PRISM), Technological University of the Shannon: Midlands Midwest, Athlone, Westmeath N37HD68, Ireland;

3. School of Intelligent Manufacturing, Huanghuai University, Zhumadian 463000, Henan, China.

*Correspondence: yuanyuan.chen@tus.ie (Y.C); 20232379@huanghuai.edu.cn (M.L).

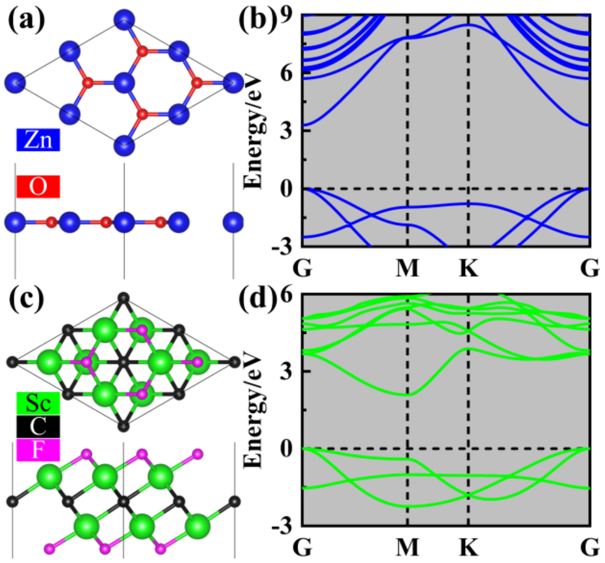

Figure S1. The 2×2×1 supercells of (a) ZnO and (c) Sc_2CF_2 monolayers. The corresponding band structures are shown in (b) and (d), respectively.

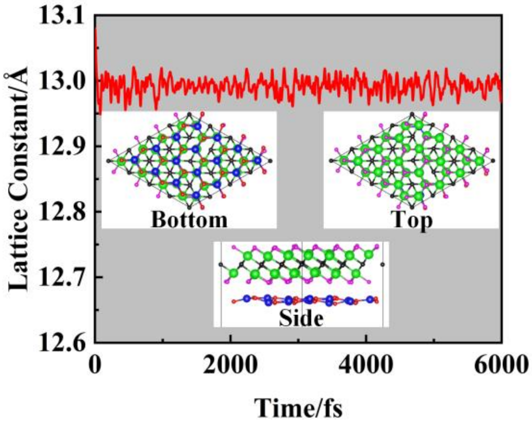

Figure S2. The evolution of lattice constant *a* for the 4×4×1 supercell of ZnO/Sc_2CF_2 heterostructure in the NPT-AIMD simulation at ambient pressure and room temperature, and the inserts are the top, side, and bottom views for its final snapshot at the end of NPT-AIMD simulation.

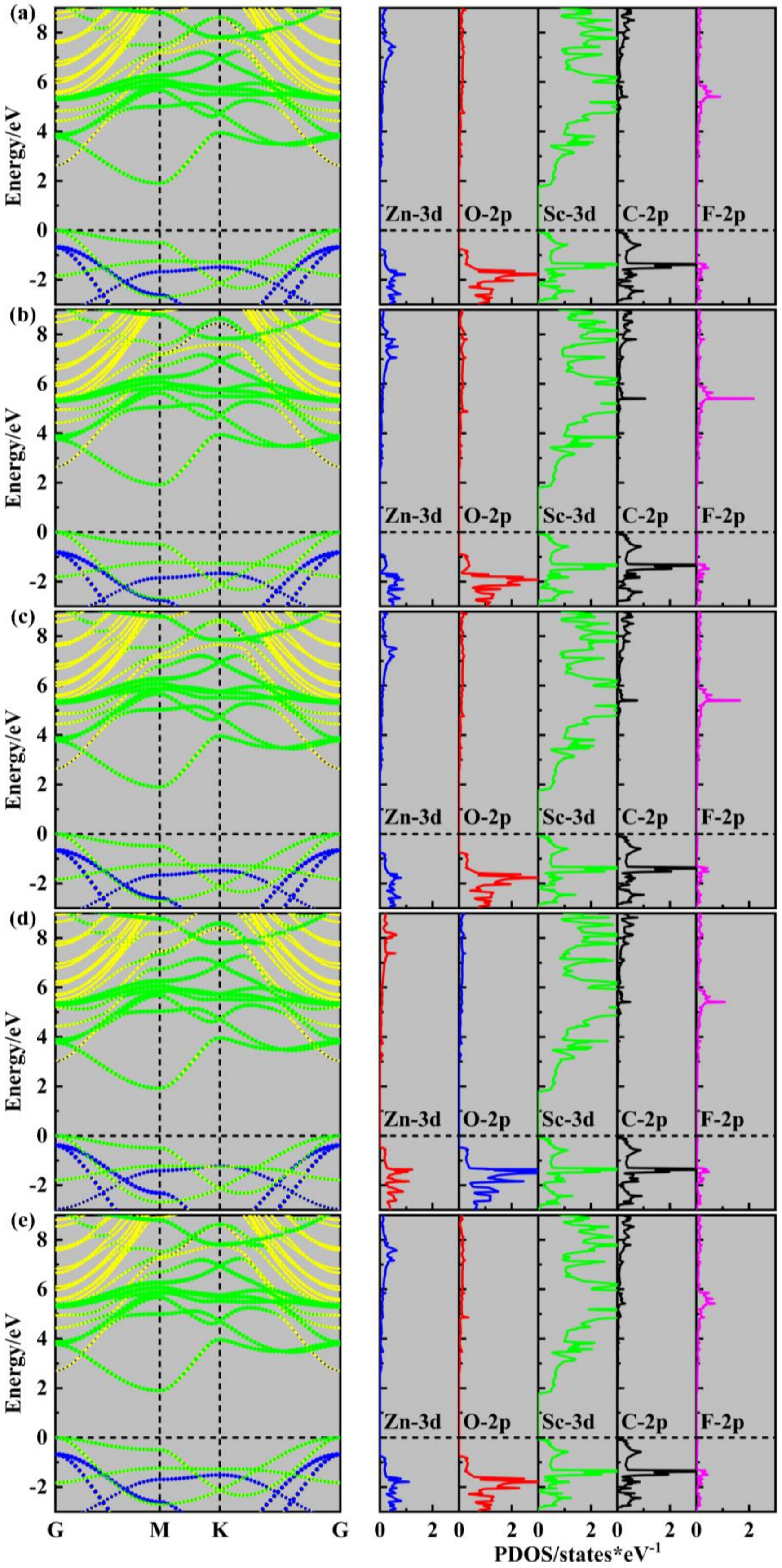

Figure S3. The projected band structures and dos of (a) SC-II, (b) SC-III, (c) SC-IV, (d) SC-V, and (e) SC-VI ZnO/Sc_2CF_2 heterostructures.

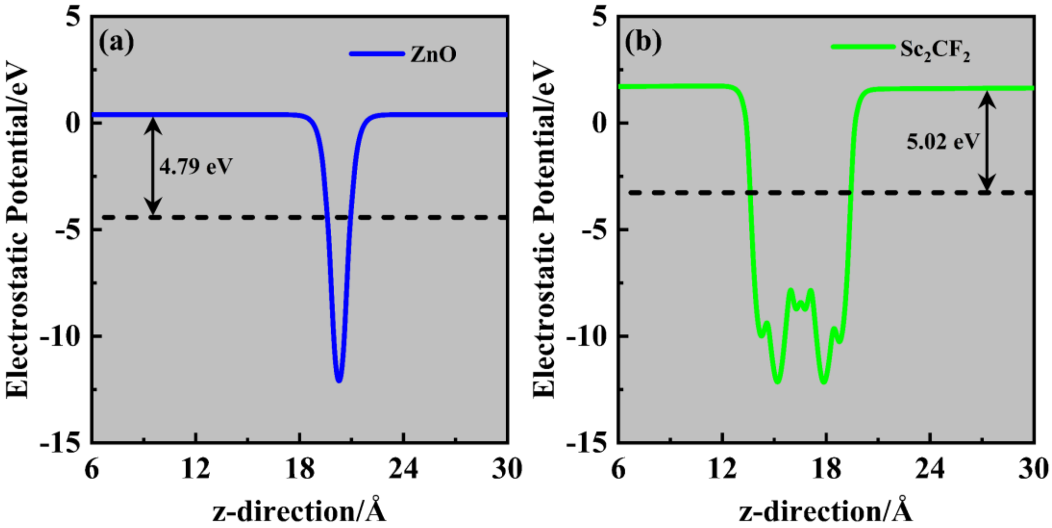

Figure S4. The potential energies of (a) ZnO and (b) Sc_2CF_2 monolayers.

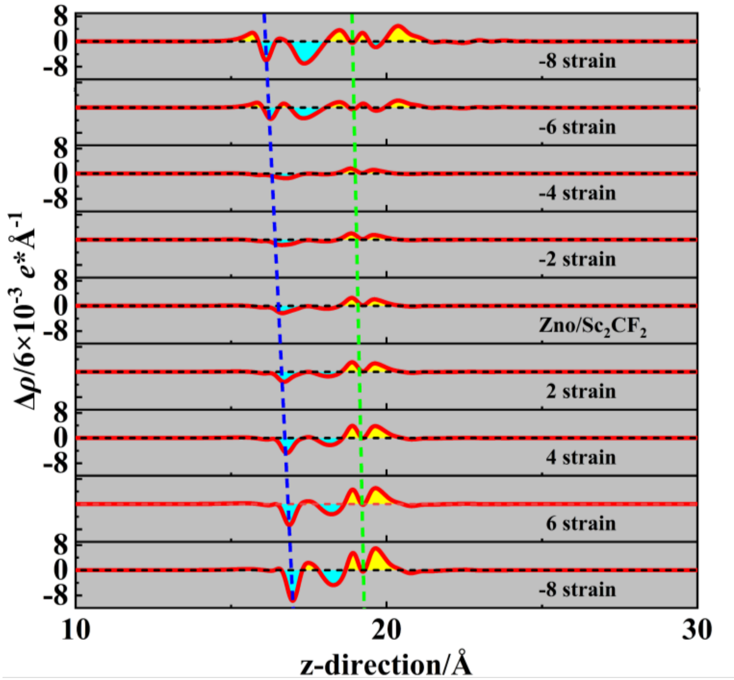

Figure S5. The planar-averaged charge density difference Δρ for the strained and free-standing ZnO/Sc_2CF_2 heterostructures.

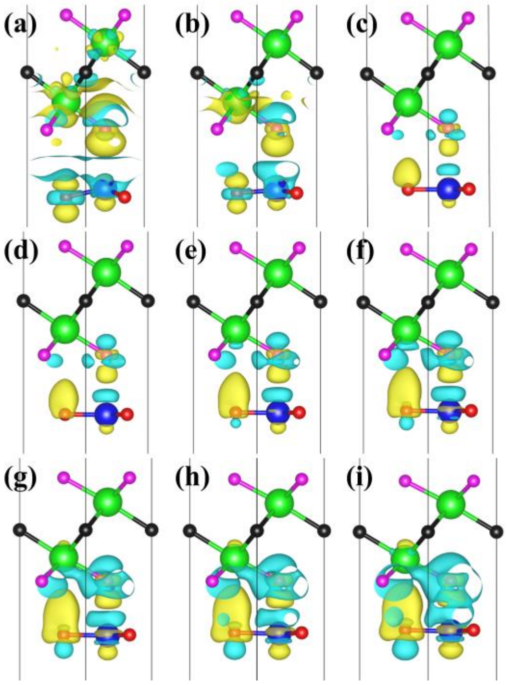

Figure S6. The charge density difference with isosurface value of 3×10^{-4} e*$Å^{-3}$ for ZnO/Sc_2CF_2 heterostructures with (a) -8%, (b) -6%, (c) -4%, (d) -2%, (e) 0%, (f) 2%, (g) 4%, (h) 6%, (i) 8% strain, respectively.

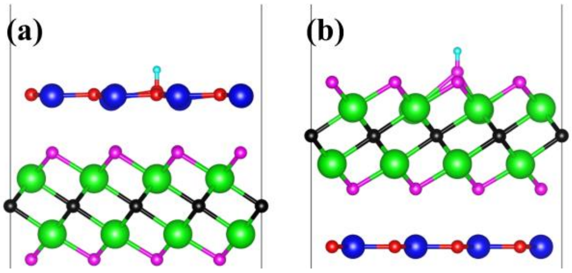

Figure S7. The top views of H-adsorption configurations on ZnO/Sc_2CF_2 heterostructure: (a) H on the ZnO side, (b) H on the Sc_2CF_2 side.

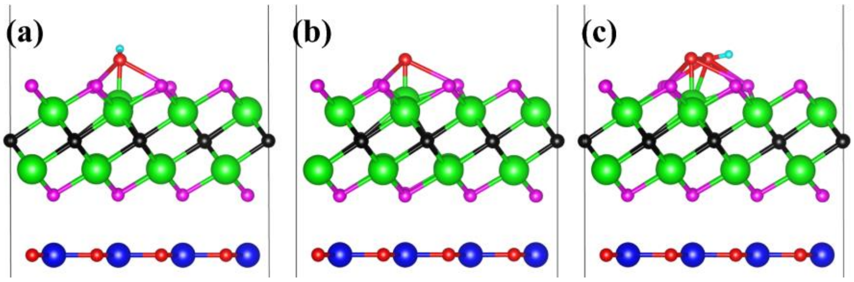

Figure S8. The top views of ZnO/Sc_2CF_2 heterostructure with absorbed intermediates: (a) OH*, (b) O*, and (c) OOH*.

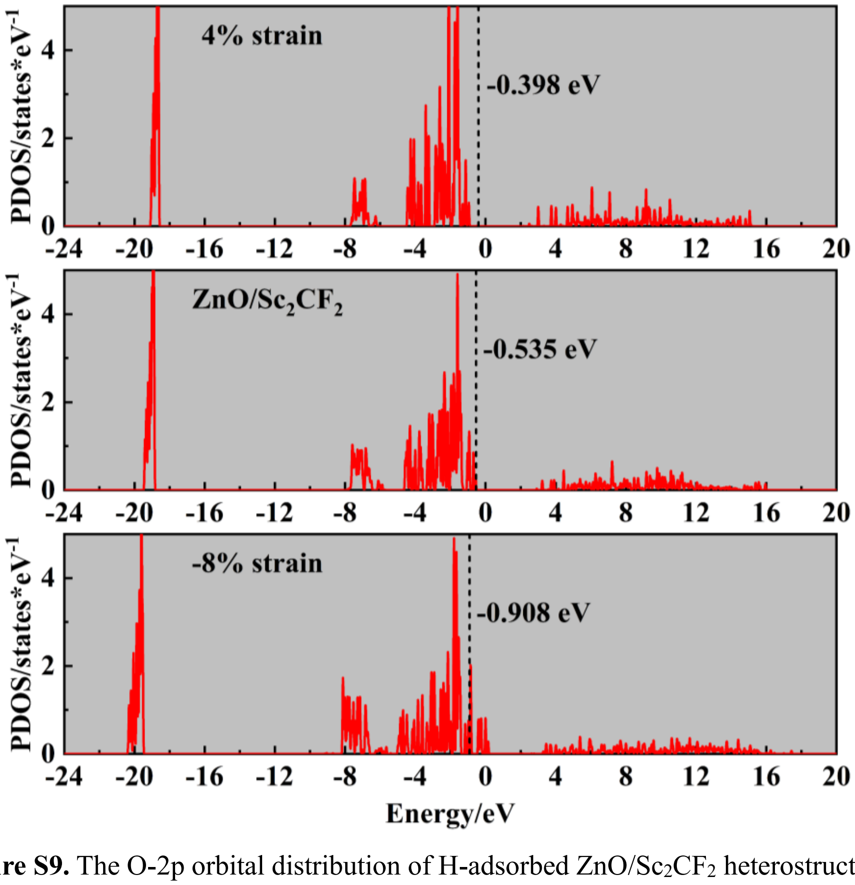

Figure S9. The O-2p orbital distribution of H-adsorbed ZnO/Sc_2CF_2 heterostructures.
